# *Yulink*, predicted from evolutionary analysis, is involved in cardiac function

**DOI:** 10.1186/s12929-020-00701-7

**Published:** 2021-01-11

**Authors:** Ming-Wei Kuo, Hsiu-Hui Tsai, Sheng-Hung Wang, Yi-Yin Chen, Alice L. Yu, John Yu

**Affiliations:** 1 Institute of Stem Cell and Translational Cancer Research, Chang Gung Memorial Hospital at Linkou, Taoyuan, 333 Taiwan; 2 Department of Pediatrics, University of California, San Diego, CA USA; 3 Institute of Cellular and Organismic Biology, Academia Sinica, Taipei, Taiwan

**Keywords:** Yulink, SERCA2, PPARγ, Ca^2+^ cycling, Cardiomyocytes

## Abstract

**Background:**

The comparative evolutionary genomics analysis was used to study the functions of novel Ka/Ks-predicted human exons in a zebrafish model. The Yulink (MIOS, Entrez Gene: 54,468), a conserved gene from zebrafish to human with WD40 repeats at N-terminus, was identified and found to encode an 875 amino acid in human. The biological function of this Yulink gene in cardiomyocytes remains unexplored. The purpose of this study is to determine the involvement of Yulink in the functions of cardiomyocytes and to investigate its molecular regulatory mechanism.

**Methods:**

Knockdown of *Yulink* was performed using morpholino or shRNA in zebrafish, mouse HL-1 cardiomyocytes, and human iPSC-derived cardiomyocytes. The expression levels of mRNA and protein were quantified by qPCR and western blots. Other methods including DNA binding, ligand uptake, agonists treatment and Ca^2+^ imaging assays were used to study the molecular regulatory mechanism by Yulink. Statistical data were shown as mean ± SD or mean ± standard error.

**Results:**

The knockdown of y*ulink* with three specific morpholinos in zebrafish resulted in cardiac dysfunctions with pericardial edema, decreased heart beats and cardiac output. The *Yulink* knockdown in mouse HL-1 cardiomyocytes disrupted Ca^2+^ cycling, reduced DNA binding activity of PPARγ (peroxisome proliferator-activated receptor gamma) and resulted in a reduction of *Serca2* (sarcoplasmic reticulum Ca^2+^ ATPase 2) expression. Expression of *Serca2* was up-regulated by PPARγ agonists and down-regulated by *PPAR*γ-shRNA knockdown, suggesting that Yulink regulates SERCA2 expression through PPARγ in mouse HL-1 cardiomyocytes. On the other hand, *YULINK, PPARγ* or *SERCA2* over-expression rescued the phenotypes of *Yulink* KD cells. In addition, knockdown of *YULINK* in human iPSC-derived cardiomyocytes also disrupted Ca^2+^ cycling via decreased SERCA2 expression.

**Conclusions:**

Overall, our data showed that *Yulink* is an evolutionarily conserved gene from zebrafish to human. Mechanistically Yulink regulated *Serca2* expression in cardiomyocytes, presumably mediated through PPARγ nuclear entry. Deficiency of *Yulink* in mouse and human cardiomyocytes resulted in irregular Ca^2+^ cycling, which may contribute to arrhythmogenesis.

## Introduction

Through comparison of the human and mouse/rat genomic sequences, Nekrutenko et al. had reported that nucleotide synonymous substitutions occurred much more frequently than nonsynonymous ones in coding regions [22]. It was predicted 13,711 novel exons that were present in both the rodent and human genomes, but the predicted transcripts remained to be validated and their biological functions remained to be demonstrated [20, 21]. Since 4768 of these predicted new exons had already been recognized as genes or pseudogenes, we used the remaining 8943 potential novel human exons to search for zebrafish orthologs in a zebrafish database (http://www.sanger.ac.uk/Projects/D_rerio/). Previously, we reported that 308 zebrafish orthologs displayed tissue- and/or developmental-specific expression [17]. In this early study, by a reverse screening process involving genetic knockdown (KD), a conserved gene, designated as *Yulink*, was identified, cloned and functionally characterized.

Here, we demonstrated that y*ulink* promoted cardiac dysfunction in zebrafish hearts, and genetic knockdown resulted in pericardial edema, decreased beating rate and cardiac output. Knockdown of *Yulink* in mouse and human iPSC-derived cardiomyocytes disrupted Ca^2+^ cycling, reduced the DNA binding activity of PPARγ, and resulted in a reduction of Serca2 expression. Expression of *Serca2* was up-regulated by PPARγ agonists and down-regulated by *PPAR*γ-shRNA knockdown, suggesting that Yulink regulates SERCA2 expression through PPARγ in mouse HL-1 cardiomyocytes. Therefore, enhancement of nuclear PPARγ activity may provide a mechanistic explanation for the involvement of Yulink in the regulation of *Serca2* expression. Finally, knockdown of *YULINK* in human iPSC-derived cardiomyocytes also disrupted Ca^2+^ cycling via decreased SERCA2 expression. This *Yulink* was also found in fly as *mio* (Gene ID: 33399) and required for the maintenance of the meiotic cycle and oocyte identity [12]. Later, it was found as a subunit of GATOR2 complex proteins in HEK-293T cells and inhibition of GATOR2 suppressed mTORC1 signaling and GATOR2 negatively regulated GATOR1 [2]. Therefore, the *Yulink* is an evolutionarily conserved gene with diverse functions.

## Materials and methods

### Bioinformatics analysis for Yulink

Protein domains on Yulink were analyzed by SMART server (http://smart.embl-heidelberg.de) using the amino acid sequences. Alignment of protein sequences and phylogenetic tree were performed using the CLUSTALW combined with ETE3 tools on GenomeNet server (https://www.genome.jp) with default parameters.

### Animals

Breeding and maintenance of TL strain zebrafish, as well as collection and staging of embryos, were performed in accordance with standard procedures [35] and approved by the Academia Sinica Institutional Animal Care and Utilization Committee. Certain embryos were reared in zebrafish egg water [35], and treated with 0.003% 1-phenyl-2-thiourea to inhibit pigmentation. Developmental times refer to hours (hpf) or days (dpf) post-fertilization.

### In situ hybridization

Embryos were fixed in 4% paraformaldehyde buffered with 1 × phosphate-buffered saline at 4 °C overnight, and proceeded to hybridized with DIG-labeled RNA antisense or control sense probes of *yulink*, embryos were incubated with anti-Dig antibody conjugated to alkaline phosphatase, and developed with NBT-BCIP reagents (Roche, Germany).

### Morpholino (MO) knockdown

Zebrafish embryos were obtained by natural mating and microinjected with morpholino (MO) before 4-cell stage. Three different MO antisense oligonucleotides, MO (5′-GGCAGGACAGTGGCTTGTTCAGTGC-3′), MO-splicing site (5′-AGTGCCTGAGGAACCAATCGTTATT-3′) and MO-start site (5′-CTGGCTTATAGCCGCTCGACATGGC-3′) were designed, that targeted specifically against the 5′ untranslated region (UTR), the splicing site and the start site of the yulink gene, respectively.

In addition, the sequence of the 5 bp mismatch negative control MO (*yulink-*5mmMO) was as follows: 5′-GGCtGcACAGTcGCTTcTTCAcTGC-3′. For the experiment, embryos injected with *yulink-*5mmMO were considered as negative control. Embryos positioned in an agarose injection chamber were injected with MO in 4.6 nl using a Narishige micromanipulator and needle holder (Narishige, Japan). The phenotype was observed using a dissecting microscope (MZ-FLIII, Leica Microsystems, Germany). Images were captured with a digital camera (SPOT, DIAGNOSTIC Instruments, USA). For hemodynamic assay, images of *yulink* KD morphant and WT hearts were dynamically monitored and captured using a dissecting microscope and digital camera at 2 dpf. Heart rate, long and short axis length of ventricle were measured from dynamic heart images. Cardiac output value was enumerated with the following formula: heart rate × (largest ventricle volume − smallest ventricle volume).

### qPCR for zebrafish

Total RNA was extracted from adult zebrafish tissues using Tri-reagent (Sigma, St. Louis, MO, USA). Reverse transcription was performed using the Superscript pre-amplification system (Gibco BRL, USA) as described in the manufacturer’s instructions. The cDNA products were amplified by PCR with specific primer sets for *yulink* or *β-actin*. The forward primer of zebrafish *yulink* was 5′-GGAACCATGTGCTGGCTGGAGG-3′ and the reversed primer, 5′-TGACTGAACCCACGGCCCTG-3′. The *β-actin* forward primer was 5′-TCACACCTTCTACAACGAGCTGCG-3′ and the reversed primer, 5′-GAAGCTGTAGCCTCTCTCGGTCAG-3′.

### Specificity of yulink gene MO

For in vivo experiments, the *yulink* 5′-UTR (46 bp in length) and its partial coding region were amplified by PCR with specific primers (5′-TCTCGAGCTCAAGCTGTTTGCACGTCAAATCTGTCA-3′ and 5′-GCAGAATTCGAAGCTCAAACACTCTGGCTCATGTTT-3′), and the amplicon was ligated into a HindIII-digested pEYFP-N1 plasmid using an In-Fusion HD Cloning Kit (Clontech, USA) to generate pYulink-EYFP. Embryos were injected with pYulink-EYFP plasmid (100 pg/embryo) alone or co-injected with pYulink-EYFP plasmid (100 pg/embryo) and either y*ulink*-MO or *yulink*-5mmMO (4.6 ng/embryo). The number of embryos expressing YFP was determined at 1 dpf.

### *Yulink* and *PPARγ* KD in mouse HL-1 cardiomyocytes

A pGIPZ lentiviral shRNAmir vector expressing a short hairpin RNA targeting *Yulink* (V2LMM_11104, mouse *Yulink*-shRNA) and a non-targeting control shRNA vector (Ctrl vector, RHS4346) were purchased from Open Biosystems (Huntsville, AL, USA).The TRCN0000001657 (*PPARγ-*shRNA) clone was obtained from the National RNAi Core Facility at the Institute of Molecular Biology (Academia Sinica, Taipei, Taiwan). HL-1 (mouse cardiac muscle) cells were obtained from Dr. W. Claycomb (Louisiana State University Medical Center, New Orleans, LA, USA), and were grown as previously described [32]. Stable KD cell lines were generated by lentivirus applied to HL-1 cells with 8 μg∕ml of polybrene (Sigma, Germany). Cells were then selected using media containing puromycin (2 μg∕ml, Sigma). RNA was isolated from cells treated with *Yulink*-shRNA, *PPARγ-*shRNA or Ctrl vector using the Quick-RNA MiniPrep Kit (Zymo Research, USA), according to the manufacturer’s instructions. The isolated RNA was reverse transcribed with the *ReverTra Ace qPCR* RT Kit (Toyobo, Japan).

The mRNA expression levels of *Yulink*, *PPARγ, Serca2a* and *GAPDH* were measured by qRT-PCR using a Roche Lightcycler 480 (Roche, Germany). The *Serca2a* primers was designed with unique sequence at 3′-UTR and not expressed in *Serca2b*. The final qRT-PCR volume in each well was 20 μl and contained 10 μl of 2× THUNDERBIRD SYBR *qPCR* Mix (Toyobo, Japan), 10 ng of cDNA, and 50 nM of gene specific primer pairs. The primers used were as follows: *Yulink*-F: CAGAGTGGCATTCGCTTGTA; *Yulink*-R: TCATTTCATTGGTCAGCTTTTC; *PPARγ*-F: GAAAGACAACGGACAAATCACC; *PPARγ*-R: GGGGGTGATATGTTTGAACTTG; *Serca2a-F*: CCTCCAGTCCTAACTTCAGTTGTT; *Serca2a-R*: CTGTCTACTGCTTCTGACTTCATTAAA; *GAPDH*-F: GGTCCTCAGTGTAGCCCAAG; and *GAPDH*-R: AATGTGTCCGTCGTGGATCT. Expression of mRNA was normalized to *GAPDH* in the same sample.

### Western blot

Lysates of HL-1 cells treated with *Yulink*-shRNA, *PPARγ*-shRNA or Ctrl vector were isolated using RIPA reagent or NE-PER Nuclear and Cytoplasmic Extraction Reagents (Thermo Fisher Scientific, USA). Protein content was quantified with the BioRad DC Protein Assay (BioRad Laboratories, USA). For Western blot, protein samples were separated by 4–12% SDS-PAGE and transferred to a PVDF membrane and probed with an appropriate primary antibody at 4 °C overnight. Antibodies were acquired from the following companies: α-Yulink (1G3) from Abnova (Taiwan), α-GAPDH (GTX100118), α-PPARα (GTX28934), α-PPARδ (GTX113250) and α-Histone H1 (GTX114462) from GeneTex (USA), α-SERCA2 (sc-8095) from Santa Cruz Biotechnology (USA), and α-PPARγ (ab27649) from Abcam (UK). Blots were then incubated with HRP-conjugated secondary antibodies (1:5000; GeneTex) for 1 h at room temperature, and proteins were detected using an ECL kit (Millipore, USA). Expression of protein was normalized to GAPDH or Histone H1 in the same sample.

### PPARγ DNA binding assays and treatment with PPAR agonists

PPARγ DNA binding activity was measured using a PPARγ transcription factor assay kit (Cayman Chemical, USA). Briefly, 10 μg of extracted nuclear proteins were added to wells containing immobilized dsDNA sequences corresponding to the peroxisome proliferator response element. Bound PPARγ was detected by the addition of specific antibodies against PPARγ. Relative PPARγ DNA binding activity was determined by normalizing the measurements obtained for cells transfected with *Yulink*-shRNA to those obtained for cells treated with Ctrl vector. Rosiglitazone (Santa Cruz), pioglitazone (Sigma), GW7647 (Sigma), and GW0742 (Sigma) were stored in the dark at − 20 °C. Working solutions were prepared by diluting the stock solution in media. Stable cells transfected with *Yulink*-shRNA or Ctrl vector were treated with or without agonist for 6 h, 12 h, or 2 days. RNA was subsequently isolated from cells using Quick-RNA MiniPrep (Zymo Research, USA), according to the manufacturer’s instructions.

### 15-Deoxy-Δ^12,14^-prostaglandin J2 uptake assay

HL-1 cardiomyocytes were incubated in medium containing 1 μM 15-deoxy-Δ^12,14^-prostaglandin J2-biotin (15d-PGJ2-Biotin) as a PPARγ ligand for 3 h, followed by PBS washing and trypsinization. After fixation with 4% PFA and staining with streptavidin-Alexa Fluor 647, the signals were analyzed by flow cytometry and immunofluorescence microscope. Approximately 10,000 cells were included in each sample for flow cytometry. The nuclei were stained with Hoechst 33342 in the immunofluorescence staining.

### Differentiation of human iPSC to cardiomyocytes

Human iPSCs were split at 1:12 ratio using 0.5 mM EDTA in PBS and grown for four days, when cells reached 85% confluence. Medium was changed to cardiomyocyte differentiation medium CDM3, which consisted of RPMI1640 basal medium, 500 μg/ ml human albumin, and 213 μg/ml l-ascorbic acid-2 phosphate [4]. Then medium was changed every other day. For day 0–2, medium was supplemented with 6 μM GSK-3 inhibitor CHIR99021. Afterword, medium was changed to CDM3 containing 2 μM Wnt signaling inhibitor Wnt-C59 on day 2–4 [4]. Starting from day 7, contracting cardiomyocytes were observed.

### *YULINK* KD in human iPSCs derived cardiomyocytes

A pGIPZ lentiviral shRNAmir vector expressing a short hairpin RNA targeting *YULINK* (V3LHS_374795, human *YULINK*-shRNA) and a non-targeting control shRNA vector (Ctrl vector, RHS4346) were purchased from Open Biosystems (Huntsville, AL). *YULINK* KD cells were generated by lentivirus applied to human iPSCs derived cardiomyocytes with 8 μg∕ml of polybrene (Sigma, Germany).

### Ca^2+^ imaging

The control and *Yulink* KD cardiomyocytes were seeded in Matrigel-coated 8-well Lab Tek II chambers (Nalge Nuc international, Rochester, NY, USA). Cells were recovered after two days and loaded with 5 μM Rhod-2 AM (Invitrogen) in Tyrode’s solution for 15 min at 37 °C as described by the manufacturer’s protocol. Ca^2+^ imaging was conducted using a Leica SP8 confocal microscope (Wetzlar, Hesse, Germany). Spontaneous Ca^2+^ transients of single beating cardiomyocyte were obtained using a time-lapse line scanning recording mode (512 pixels × 1920 lines) under 40 × objective at room temperature, and the raw data was analyzed using Leica LAS X program. Ca^2+^ signal was normalized to the intracellular basal line (F_0_), and the transient amplitude was expressed as F/F_0_. In addition, the τ (Tau) is the exponential decay of time constant for the speed of calcium uptake, which is commonly used as one method for characterizing the speed of Ca^2+^ recovery. A large τ value indicates a longer recovery time to baseline; the units of τ are in time (seconds). The time constant, Tau, represents the elapsed time required for the calcium amount to decay to 1/e = 36.8% of the original value.

### Over-expression of *YULINK*, *PPARγ* or *SERCA2* in mouse HL-1 cardiomyocytes

To generate the over-expression of *YULINK*, *PPARγ* or *SERCA2* plasmids, the PCR products of their full-length coding regions were generated using pEF1-Flag/His-YULINK vector or cDNA from human iPSC-derived cardiomyocytes. The specific primer sets were 5′-tagagctagcgaattcatgagcggtaccaaacctgatattt-3′ and 5′-tcgcggccgcggatccttatggctggacagtctctgcaggta-3′ for *YULINK*; 5′-tagagctagcgaattcatgaccatggttgacacagag-3′ and 5′-tcgcggccgcggatccctagtacaagtccttgtagatctc-3′ for *PPARγ*; 5′-tagagctagcgaattcatggagaacgcgcacaccaag-3′ and 5′-tcgcggccgcggatccttactccagtattgcaggttcc-3′ for *SERCA2*. Then these PCR products were inserted into the lentiviral pCDH plasmid containing BFP (System Biosciences, linearized by EcoRI and BamHI double digestion) using an In-Fusion HD Cloning Kit (Clontech). All constructs were checked using Sanger sequencing (Genomics BioSci & Tech). Over-expression of *YULINK* and *PPARγ* plasmids were transfected into *Yulink* KD cells using lipofectamine 3000 transfection reagent (Thermo Fisher Scientific). Over-expression of *SERCA2* plasmid was electroporated into *Yulink* KD cells using Neon electroporation system (ThermoFisher).

### Statistical analysis

Some data were shown as the mean ± SD whereas other values were shown as mean ± standard error. A *p *value or post-hoc *p* value < 0.05 was considered statistically significant.

## Results

### Structure of Yulink

**Fig. 1:**
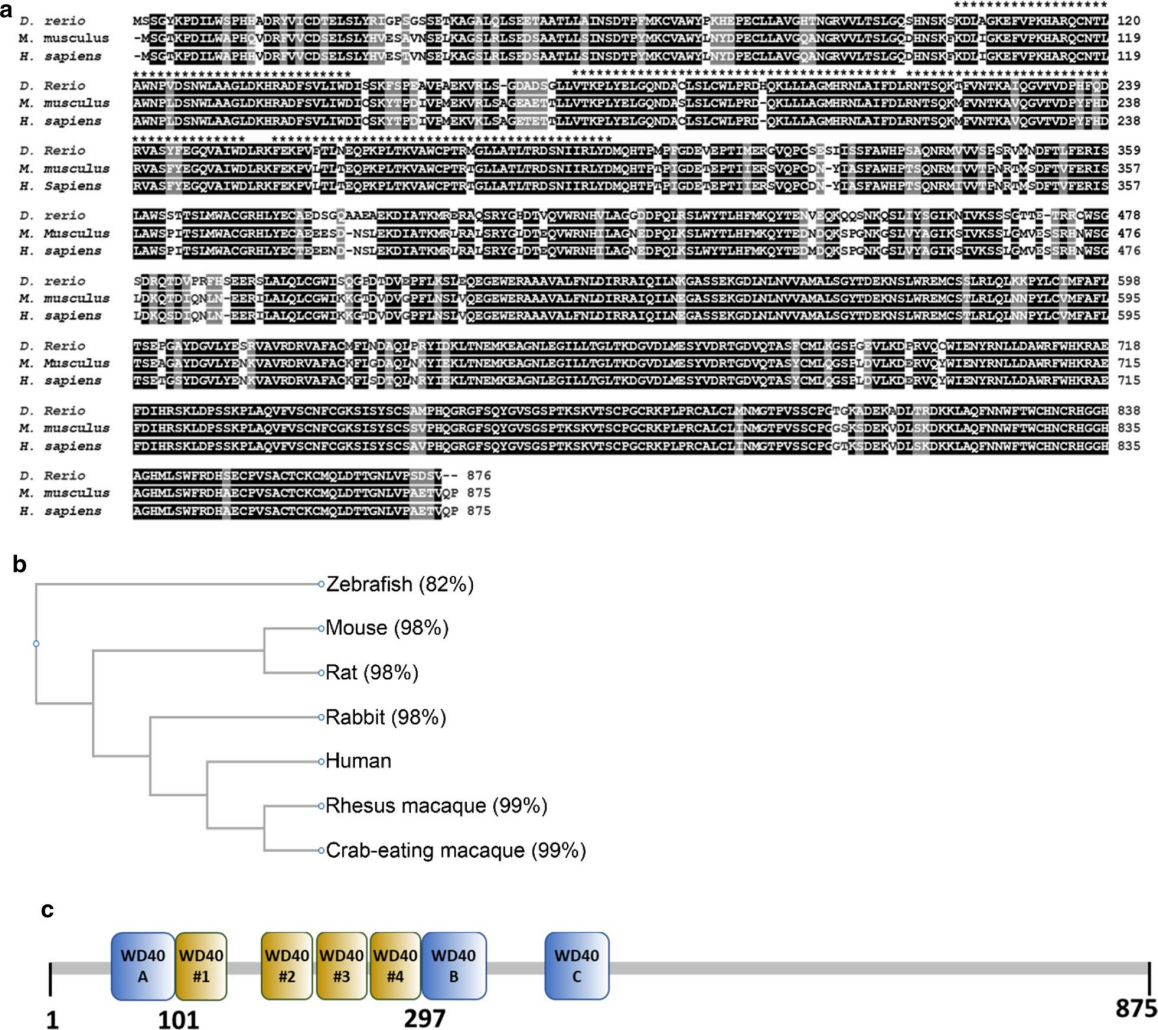
Structural features of Yulink protein. **a** The multiple sequence alignment analysis was performed with Clustal Omega software (https://www.ebi.ac.uk/Tools/msa/clustalo/). The amino acid identity between human (NP_061878.3) and zebrafish (NP_958490.1) was 82%, and between human and mouse (NP_663349.2) was 98%. “Dark gray color” means that the residues in that column are identical in all sequences in the alignment. “Light gray color” means that the residues were conserved with strongly similar properties. *** refers to WD40 repeats. **b** Classification and similarity of homologs of Yulink proteins. The classification of these homologs of Yulink was shown with a phylogenetic tree. The percentages of identities in amino acid sequences for homologous proteins were compared with human Yulink. **c** WD40 repeats within YULINK predicted by four computer servers. The prediction for WD40 repeats were based on the CDD, UniProt, SMART, and InterPro computer servers. WD40 #1 to #4 were the common results based on prediction using the four computer servers. The WD40 A and B were identified through the annotation of the CDD server, whereas WD40 C was found with the UniProt server

Previously, we performed comparative evolutionary genomics analysis to study the functions of 13,711 novel Ka/Ks-predicted human exons using zebrafish (*Danio rerio*) as a model organism [17, 20–22]. Through a reverse screening process involving genetic knockdown (KD), a novel gene, designated as *Yulink* (*MIOS*, Entrez Gene: 54468), which encodes an 875 amino acid protein with WD40 repeats at N-terminus in human and mouse (Fig. 1a). The similarities of protein sequences among Yulink genes of common experimental animals were analyzed by multiple sequence alignment using CLUSTALW (https://www.genome.jp). The classification of these homologs of Yulink was shown with a phylogenetic tree (Fig. 1b). When compared with the protein sequence of human YULINK, the phylogenetic tree indicated that YULINK proteins of rhesus and crab-eating macaque were the most similar homologs, with 99% of identities in amino acid sequences (Fig. 1b). The most dissimilar homolog was yulink from zebrafish, which exhibited 82% identities (Fig. 1b). The identities for homologs of Yulink from mouse, rat and rabbit were all approximately 98% (Fig. 1b). These analyses indicated that the *Yulink* gene is highly conserved in diverse species of animal, implying that the *Yulink* may have potentially conserved functions from zebrafish to human.

The 3D structure of YULINK protein (Gene ID: 54468) is currently unavailable. A WD40 repeat is traditionally defined as a structural motif with about 40 amino acids composed of four β-strands which is often terminated with Trp-Asp (WD) sequence [37]. In addition, seven WD40 repeats can further be assembled to form a WD40 domain [37]. Such structural features were conventionally analyzed with computer servers. Currently, to explore these structural features, the following four database servers are available for annotation: (i) Conserved Domain Database (CDD, https://www.ncbi.nlm.nih.gov/cdd), (ii) UniProt (https://www.uniprot.org), (iii) SMART (http://smart.embl-heidelberg.de), and (iv) InterPro (https://www.ebi.ac.uk/interpro) servers.

It was found that with these four computer servers, the region of AA 101 to 297 of YULINK displayed clearly four conserved WD40 repeats (i.e. WD40 #1–#4 in Fig. 1c, yellow). However, two additional candidates for WD40 repeats were also revealed by the Conserved Domain Database (WD40 A and B in Fig. 1c, blue). Furthermore, the UniProt server had annotated one additional WD40 repeat by (WD40 C in Fig. 1c, blue). These analyses thus suggest that YULINK may contain four conventional WD40 repeats and three additional potential candidates,

For further validation, we examined the secondary structure of YULINK in details. Since WD40 repeat is known to possess secondary structure as four β-strands, the distribution of β-strands in YULINK was analyzed with the Jpred4 (http://www.compbio.dundee.ac.uk/jpred) (Additional file 1: Fig. S1). As shown, the first half of YULINK contains seven WD40 repeats (#1–#4 and A–C) as predicted by four computer servers to possess the prerequisite features. The distribution of secondary structure as analyzed by Jpred4 server further confirmed that each predicted WD40 repeat displayed three to four tandem β strands (Additional file 1: Fig. S1); these structural features are reminiscent of the characteristics for typical WD40 repeats. In conclusions, YULINK has four conserved WD40 repeats and three additional potential WD40 repeat candidates.

### Specificity of *yulink*-MO knockdown (KD) in zebrafish morphants

**Fig. 2:**
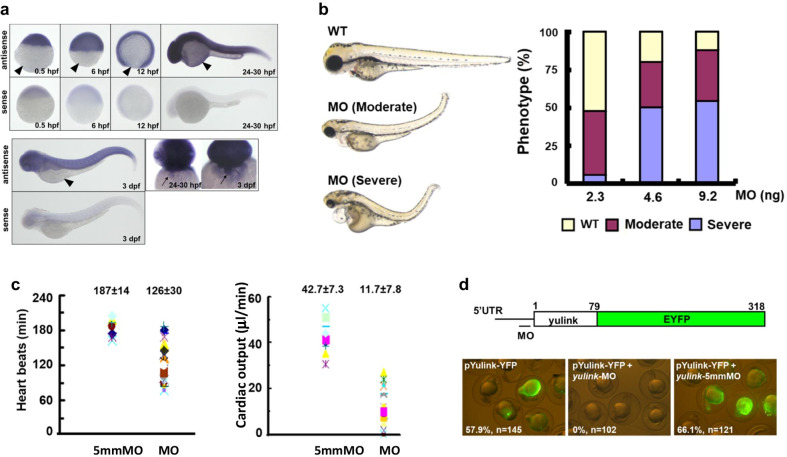
Characterization of *yulink*-MO KD zebrafish morphants. **a** Expression of *yulink* in zebrafish using whole-mount in situ hybridization. Embryos were fixed overnight at 4 °C in 4% paraformaldehyde buffered with 1 × phosphate-buffered saline (PFA/PBS). After hybridization with Dig-labeled antisense or control sense RNA probes of *yulink*, embryos were incubated with anti-Dig antibody conjugated to AP and developed with NBT-BCIP reagents. The *yulink* was expressed in whole zebrafish embryo ubiquitously from 0.5 hpf (zygote stage) to 3 dpf (larval stage) with lateral overview. The black arrows point to heart regions at 24–30 hpf and 3 dpf with ventral overview. The black arrowheads point to yolk of embryos, hour post-fertilization (hpf); day post-fertilization (dpf). **b** After injection with *yulink-*MO to knockdown *yulink* expression, the *yulink* KD morphants presented with small eyes, a small head, abnormal blood circulation, and pericardial edema at 3 dpf. Increasing the *yulink*-MO dosage from 2.3 ng (n = 119) to 4.6 ng (n = 119 or 9.2 ng (n = 156) caused the proportion of severely affected embryos with pericardial edema to increase from 6 to 54% (purple). **c** Hemodynamic changes. *Yulink* KD morphants had a slower heart rate and reduced cardiac output, as compared to WT embryos at 2 dpf (** *p* < 0.01, Student’s *t* test). **d** Diagram of the EYFP-fusion construct with the *yulink* 5′-UTR region and partial coding region (amino acids 1–79). The position of the corresponding MO binding site is indicated by a bar. To demonstrate the specificity of the *yulink*-MO, embryos were injected with pYulink-EYFP plasmid (100 pg/embryo) alone, or together with *yulink*-MO or the mismatch control, *yulink*-5mmMO (4.6 ng/embryo). About 58% of embryos injected with pYulink-EYFP plasmid alone exhibited fluorescence at 1 dpf, while fluorescence was absent in embryos co-injected with *yulink*-MO. In contrast, co-injection with *yulink*-5mmMO did not affect EYFP expression

The *yulink* was expressed in whole zebrafish embryo ubiquitously from 0.5 hpf (zygote stage) to 3 dpf (larval stage) and expressed in heart region starting at 24–30 hpf (Fig. 2a). To investigate the biological function of the yulink gene, zebrafish embryos before the 4-cell stage were injected with the antisense oligonucleotide *yulink*-MO, which targeted against to 5′-UTR, to knockdown gene function. *Yulink* KD morphants showed small eyes, a small head, and marked pericardial edema, as compared to the wild type at 3 dpf (Fig. 2b). Increasing the *yulink*-MO amount from 2.3 to 9.2 ng caused the proportion of the severely affected embryos with pericardial edema from 6 to 54% (Fig. 2b). We had also constructed two other MOs, which targeted against splicing site and translational start site of the yulink gene, respectively, the results are shown in Additional file 1: Fig. S2. The abnormal phenotypes (e.g. small eyes, a small head, marked pericardial edema, etc.) after treatment of these MOs were similar to those in the morphants originally observed in Fig. 2b, at wide range dosage of MOs (1–10 ng/embryo).

In addition, severely affected embryos which showed marked pericardial edema exhibited defects in blood circulation. The *yulink* KD morphants exhibited slower heart rates (126 beats/min), as compared to mismatch control 5mmMO (187 beats/min, Fig. 2c, Additional file 1: Video). Besides, the *yulink* KD morphants also exhibited reduced cardiac output averaged 11.7 μl/min, as compared to the 5mmMO averaged 42.7 μl/min (Fig. 2c). Loss of function for *serac2a* [7] or several ion channel related genes (e.g. the “hyperpolarization activated cyclic nucleotide gated potassium channel 4”, *hcn4* [14, 29], and T-type calcium channels, *a1G, a1Ha, a1Hb, a1la* and *a1lb* [27]) were reported to reduce the heart rate. Therefore, specific qPCR primers were designed for these genes (Additional file 1: Fig. S3A). The expression levels of *serca2a* and the ion-channel related genes were found to decrease significantly in *yulink* KD morphants (orange color) after normalization with the internal control (*bactin1*) at 3 dpf (Additional file 1: Fig. S3b, c), thus consistent with the observed slower heart rates for the *yulink* KD morphants.

To further confirm the above findings were not caused by off-target effect, the 5′-UTR of *yulink* and its partial coding sequences were introduced into the pEYFP-N1 plasmid to generate the pYulink-EYFP plasmid (Fig. 2d). Approximately 58% of the embryos injected with this plasmid (100 pg/embryo) exhibited EYFP fluorescence in vivo (Fig. 2d). In a parallel co-injection with *yulink*-MO totally suppressed EYFP expression; in contrast, the mismatch control, *yulink*-5mmMO did not affect EYFP expression (Fig. 2d). To further assess the specificity of the *yulink*-MO, the ability of synthesized mouse *Yulink* mRNA to protect against the *yulink*-MO-induced changes in phenotype was determined, using the *β-gal* mRNA as an experimental control (Additional file 1: Fig. S4). After injection of *yulink*-MO into zebrafish embryos, the proportions of morphants with severe (gray color) and moderate (orange color) phenotype changes were, respectively, 65.8% and 18.6%. But, co-injection of the control *β-gal* mRNA and *yulink*-MO did not change the proportions of morphants which displayed severe and moderate phenotypes (62.2% and 15.6%); these values were similar to those observed with the injection of *yulink*-MO alone. However, co-injection with *Yulink* mRNA reduced the proportions of morphants with severe phenotype significantly to 38.7%. Importantly, the morphants with moderate or normal phenotypes were found to increase to 30.65% and 30.65%, respectively (Additional file 1: Fig. S4). These results thus implied that morphants caused by injection of *yulink*-MO could be competed (rescued) to become the morphants with less severe (moderate) and WT phenotype with the co-injection of *Yulink* mRNA. It was thus concluded that Yulink is important for the heart development and cardiac function in zebrafish; but detailed characterization … await for future electrophysiological studies.

### *Yulink* knockdown induces irregular Ca^2+^ cycling in mouse HL-1 cardiomyocytes

**Fig. 3:**
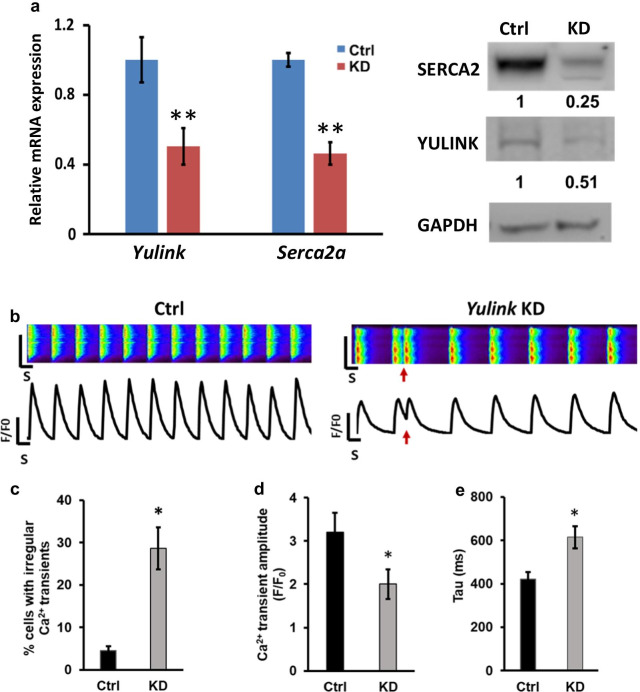
*Yulink* KD reduced *Serca2* expression and induced irregular Ca^2+^ cycling in mouse HL-1 cardiomyocytes. **a** Representative line-scan images and spontaneous Ca^2+^ transient in Ctrl and *Yulink* KD mouse HL-1 cardiomyocytes. Red arrows indicate arrhythmia-like waveforms observed in *Yulink* KD HL-1 cardiomyocytes, but not in control. **b** Quantification of percentages for control and *Yulink* KD HL1 cardiomyocytes exhibiting irregular Ca^2+^ transients (n = 40). **c** The Ca^2+^ transient amplitude of HL-1 cardiomyocytes for Ctrl and *Yulink* KD confirms *Yulink* KD HL-1 cardiomyocytes exhibits a lower Ca^2+^ transient amplitude (n = 40). **d** The time constant for Ca^2+^ decay (Tau) of HL-1 cardiomyocytes for control and *Yulink* KD show that the time constant for Ca^2+^ decay is significantly larger in *Yulink* KD than in control HL-1 cardiomyocytes (n = 40, *p < 0.05, Student’s *t* test). **e** HL-1 cardiomyocytes were subjected to *Yulink* KD using *Yulink*-shRNA, and the effects on *Yulink* and *Serca2* expression were assayed by qPCR and Western blot. Relative expression values were normalized to those of cells injected with control (Ctrl) vector (n = 3 for each group, **p < 0.01, Student’s *t* test)

The *yulink* KD morphants in zebrafish reduced the heart rates and cardiac output suggest that the Yulink may play an important role in cardiomyocytes. To investigate this possibility, mouse HL-1 cardiomyocytes were transduced with lentivirus expressing short hairpin RNA (shRNA) against *Yulink*. Quantitative PCR and western blotting analysis of the transduced cells confirmed a *Yulink* knockdown with efficiency of ~ 50% (Fig. 3a).

Calcium (Ca^2+^) plays a critical role in regulation of excitation–contraction coupling and is essential in the electrical signaling of cardiomyocytes. Abnormal Ca^2+^ cycling is linked to arrhythmogenesis, which is associated with cardiac disorders and heart failure [10]. Accordingly, we measure Ca^2+^ cycling from control and *Yulink* KD HL-1 cells using confocal fluorescence microscopy loaded with fluorescent Ca^2+^ dye Rhod-2 AM. Compared to control, *Yulink* KD demonstrated significant higher Ca^2+^ transient irregularities which may relate to triggered arrhythmia-like waveforms (Fig. 3b, red arrows); irregular Ca^2+^ transients were virtually absent in control cells (28.6% for *Yulink* KD vs. 4.6% for control, Fig. 3c).

Moreover, we also observed that the *Yulink* KD HL-1 cells exhibited defective intracellular Ca^2+^ cycling with a significantly observed reduced amplitude of Ca^2+^ transients (F/F_0_ = 1.87 ± 0.31) than in control cells (F/F_0_ = 2.89 ± 0.34) (Fig. 3d). In particular, the *Yulink* KD exhibited defective intracellular Ca^2+^ cycling with a significantly slower Ca^2+^ decay rate (615 ± 51 ms) than control (421 ± 34 ms) (Fig. 3e). These data suggest that knockdown of *Yulink* contribute to abnormal intracellular Ca^2+^ release and arrhythmogenic phenotype in mouse HL-1 cardiomyocytes.

### Over-expression of *YULINK *rescued the phenotypes of *Yulink* KD HL-1 cells

**Fig. 4:**
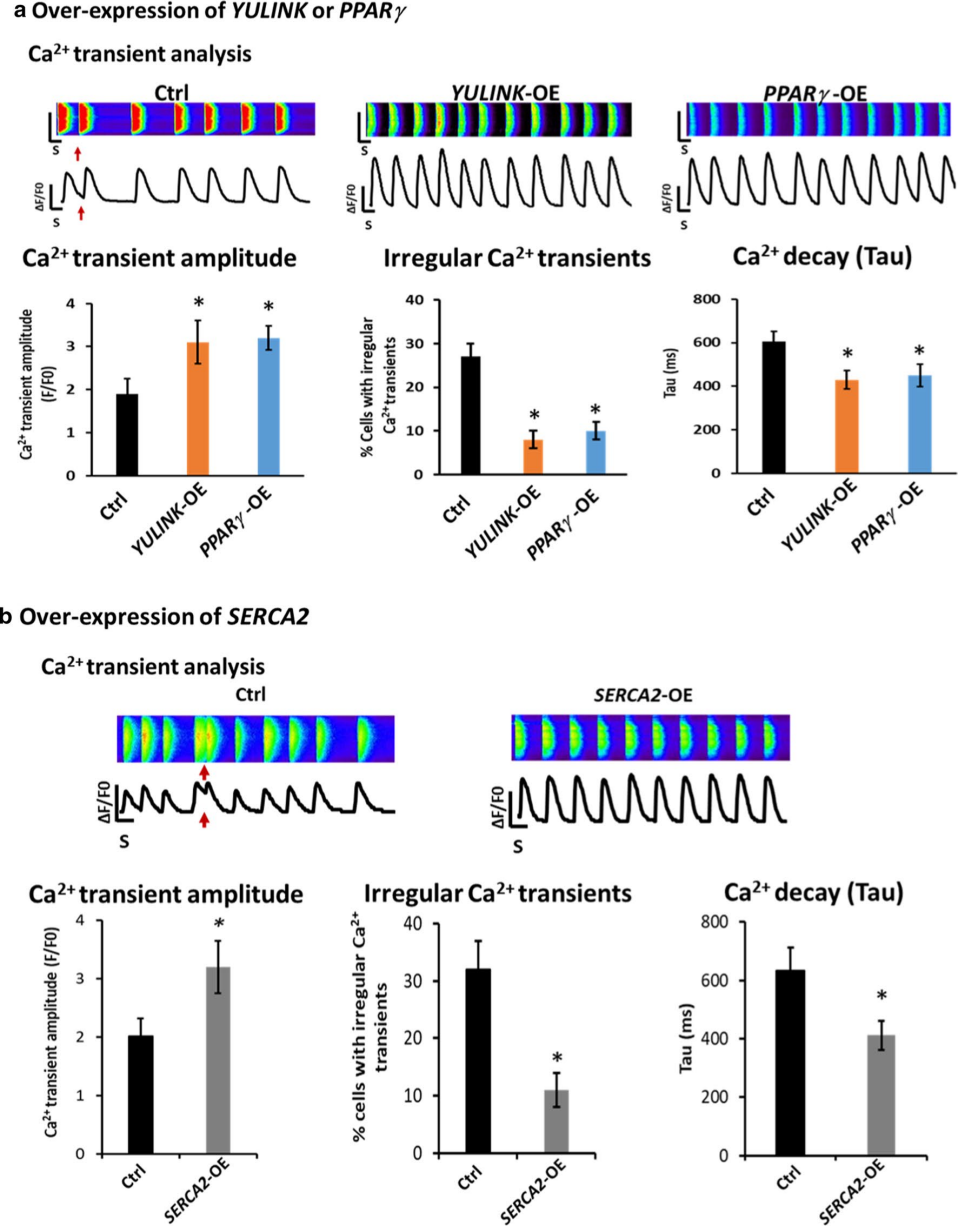
Over-expression of *YULINK, PPARγ* and *SERCA2* rescued the phenotypes of mouse *Yulink* KD HL-1 cardiomyocytes. Control (Ctrl), YULINK, PPARγ or SERCA2 plasmids (carrying BFP as indicator of expression marker) were transfected or electroporated into *Yulink* KD cells, and then analyzed for Ca^2+^ cycling using fluorescent Ca^2+^ dye, Rhod-2 AM. **a** Over-expression of *YULINK* or *PPARγ*. Representative line-scan images and spontaneous Ca^2+^ transient in *Yulink* KD mouse HL-1 cardiomyocytes. Arrhythmia-like waveforms were observed in *Yulink* KD cardiomyocytes with Ctrl vector (red arrows). After over-expression of *YULINK* (*YULINK*-OE) or *PPARγ* (*PPARγ*-OE) in *Yulink* KD cells, normal waveforms were observed. The result of Ca^2+^ spark analysis was consistent with the increase of Ca^2+^ transient amplitudes (3.1 ± 0.5 for *YULINK-*OE or 3.2 ± 0.3 for *PPARγ*-OE, vs. 1.9 ± 0.35 for Ctrl). The cells with *YULINK* or *PPARγ* over-expression also exhibited a decrease of the percentages of irregular Ca^2+^ transients (8 ± 2% for *YULINK-*OE or 10 ± 2% for *PPARγ*-OE, vs. 27 ± 3% for Ctrl). The cells with *YULINK* or *PPARγ* over-expression also exhibited a reduction of the Ca^2+^ decay (Tau) rate (430 ± 42 ms for *YULINK-*OE or 450 ± 51 ms for *PPARγ-*OE, vs. 605 ± 47 ms for Ctrl). Ctrl (black bars), *YULINK*-OE (orange bars) and *PPARγ*-OE (blue bars) (n = 40, each). *p < 0.05, Student’s *t* test. **b** Over-expression of *SERCA2.* Representative line-scan images and spontaneous Ca^2+^ transients in *Yulink* KD mouse HL-1 cardiomyocytes. Arrhythmia-like waveforms were observed in *Yulink* KD cardiomyocytes with control vector (Ctrl, red arrows). After over-expression of *SERCA2* (*SERCA2*-OE) in *Yulink* KD cells, normal waveforms were observed. The result of Ca^2+^ spark analysis was consistent with the increase of Ca^2+^ transient amplitudes (3.2 ± 0.45 for *SERCA2-*OE vs. 2.02 ± 0.3 for Ctrl). The cells with *SERCA2*-OE also exhibited a decrease of the percentages of irregular Ca^2+^ transients (11 ± 3% for *SERCA2-*OE vs. 32 ± 5% for Ctrl). The cells with *SERCA2*-OE also exhibited a reduction of the Ca^2+^ decay (Tau) rate (412 ± 50 ms for *SERCA2-*OE vs. 635 ± 78 ms for Ctrl). Ctrl vector (black bars), *SERCA2-*OE (gray bars) (n = 35, each). *p < 0.05, Student’s *t* test

In order to study whether the over-expression (OE) of *YULINK* rescued the phenotypes of *Yulink* KD HL-1 cells, control (Ctrl) or YULINK plasmid (carrying BFP as indicator of expression marker) were transfected into *Yulink* KD cells, and then Ca^2+^ cycling were analyzed using the fluorescent Rhod-2 AM dye. In Fig. 4a, the intracellular Ca^2+^ cycling waveforms became normal, when these KD cells were over-expressed with *YULINK*, as compared to *Yulink* KD cells. In addition, the results of Ca^2+^ sparks analysis were consistent with the increase of Ca^2+^ transient amplitudes (3.1 ± 0.5 for *YULINK*-OE, orange bar, vs. 1.9 ± 0.35 for Ctrl, black bar) (Fig. 4a). These cells with *YULINK*-OE also exhibited a decrease of the percentages of irregular Ca^2+^ transients (8 ± 2% for *YULINK*-OE, orange bar, vs. 27 ± 3% for Ctrl, black bar) and the reduction of the Ca^2+^ decay rate (430 ± 42 ms for *YULINK*-OE, orange bar, vs. 605 ± 47 ms for Ctrl, black bar) (Fig. 4a). The level of Ca^2+^ transient amplitudes, the percentages of irregular Ca^2+^ transients, and the Ca^2+^ decay (Tau) rate were all similar between Ctrl (Fig. 3b) and over-expression of *Yulink* cells. These data indicate that the *Yulink*-shRNA used in our studies was specific and the observed defects in cells were indeed Yulink-dependent.

### Knockdown of *Yulink* reduces *Serca2* expression in mouse HL-1 cardiomyocytes

Several studies have suggested that expression and function of *Serca2* play a major role in defective intracellular Ca^2+^ cycling [11, 26, 32]. The decreased *Serca2* expression may also contribute to mechanical failure in cardiomyocytes [24]. Therefore, it is hypothesized that *Yulink* may play a role in regulating SERCA2 expression. SERCA2a, one of the two isoforms for SERCA2, was expressed in cardiac muscle, slow-twitch skeletal muscle, and smooth muscle cells, while SERCA2b is an ubiquitous isoform expressed in muscle and non-muscle cells [25]. It was also reported that approximately 95% of the SERCA2 protein in mouse HL-1 cardiomyocytes are Serca2a [34]. To examine the *Serca2a* expression in the *Yulink* KD mouse HL-1 cardiomyocytes, we first performed quantitative PCR using specific *Serca2a* primers, designed based on a unique sequence at 3′-UTR region which were not expressed by *Serca2b*. As shown in Fig. 3a, there was significant reduction of *Serca2a* expression after *Yulink* KD by qPCR. Similarly, on Western blot analysis, there was also reduction of SERCA2 protein expression (Fig. 3a). Therefore, these results demonstrate that the expression of *Yulink* is required for *Serca2* expression. These data imply that *Yulink* may be involved in the expression of *Serca2* as well as the intracellular Ca^2+^ cycling in mouse HL-1 cardiomyocytes.

### Knockdown of *Yulink* blocks the entry of PPARγ ligands into cells and decreases PPARγ DNA binding activity in the nuclei

**Fig. 5:**
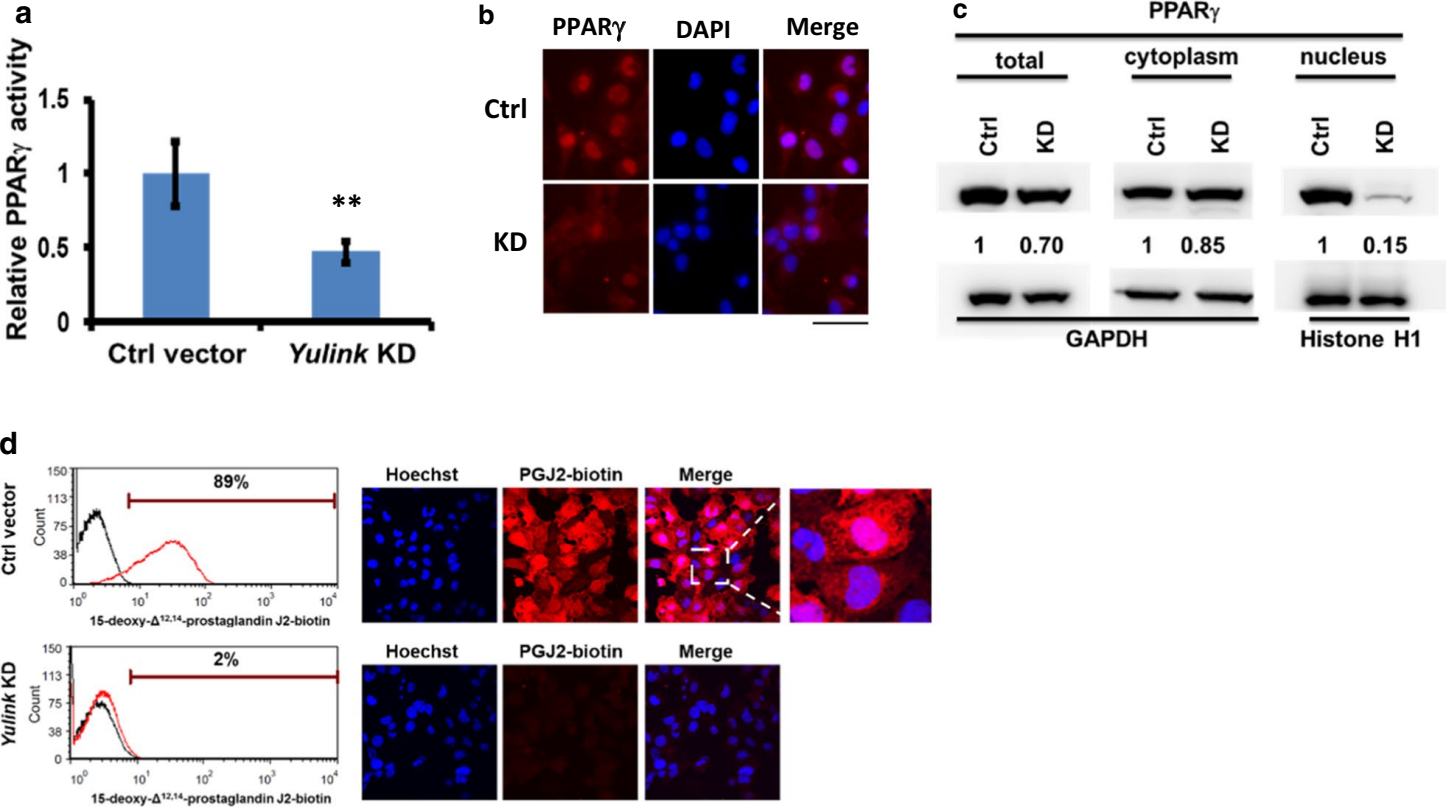
Effect of *Yulink* KD on PPARγ activity in HL-1 cardiomyocytes. **a** The DNA-binding activity of nuclear PPARγ in HL-1 cardiomyocytes was significantly reduced by *Yulink* KD using *Yulink*-shRNA (left panel); values normalized to those in cells treated with Ctrl vector (relative PPARγ activity) are shown. (n = 3, ** *p* < 0.01, Student’s *t* test). **b** The nuclear PPARγ expression level (Red) was decreased in *Yulink* KD cardiomyocytes. In the immunofluorescence assay, PPARγ was stained red, and nuclei were stained with DAPI (blue). **c** The amounts of total, cytoplasmic, and nuclear PPARγ were determined by Western blot, and normalized to an internal control (GAPDH or Histone H1) (right panel). Total PPARγ protein was decreased by 30%, while nuclear PPARγ was decreased by 85% in *Yulink* KD cardiomyocytes. **d** KD of *Yulink* decreases the uptake of the PPARγ ligand 15d-PGJ2-biotin in cardiomyocytes. Cells were incubated in 1 μM 15d-PGJ2-biotin for 3 h, and then fixed and stained with streptavidin-Alexa Fluor647 (red). Signals were detected using flow cytometry and immunofluorescence. Black lines indicate cells incubated without ligand; red lines indicate cells incubated with ligand in flow cytometric analyses. In the immunofluorescence assay, 15d-PGJ2-biotin was stained red, and nuclei were stained with Hoechst 33342 (blue). The majority of cardiomyocytes transfected with control vector took up the 15d-PGJ2-biotin (upper panel), while only 2% of *Yulink* KD cardiomyocytes took up the ligand (bottom panel)

The peroxisome proliferator-activated receptors (PPARs) are ligand-activated transcription factors of the nuclear receptor superfamily [13]; one member of this family, PPARγ has been reported to directly bind to a PPARγ response element within the *Serca2* gene proximal promoter in pancreatic islet cells [16]. We next evaluate the *PPARγ* DNA-binding activities in the *Yulink* KD cardiomyocytes using the immobilized dsDNA corresponding to the peroxisome proliferator response element. As showed in Fig. 5a, the *PPARγ* DNA-binding amount of *Yulink* KD HL-1 was significantly decreased to 47% compared to control cells. Consistent with these findings, we also found that the nuclear PPARγ was decreased in *Yulink* KD cardiomyocytes by immunofluorescence staining (Fig. 5b). Western blot analysis also showed that the nuclear PPARγ protein level was decreased by 85% in *Yulink* KD cardiomyocytes, respectively (Fig. 5c). In this assay, the decrease of PPARγ in lysate of *Yulink* KD cell nuclei also reflected a lower DNA binding activity. The *Yulink* KD resulted in diminished *PPARγ* DNA-binding and nuclear PPARγ protein level in HL-1 cardiomyocytes suggest that Yulink may involve in the nuclear import of PPARγ.

The ligands for PPARγ are known to include fatty acids, arachidonic acid metabolites, and thiazolidinediones, such as rosiglitazone and pioglitazone [28, 31]. In addition, 15-deoxy-Δ^12,14^-prostaglandin J2 (15d-PGJ2) has been reported as an endogenous ligand for PPARγ activation [9, 15]. Therefore, we used the biotinylated 15d-PGJ2 to characterize the ligand uptake in *Yulink* KD cardiomyocytes by flow cytometry and immunofluorescence (Fig. 5d). The results showed that 89% of the fluorescent 15d-PGJ2-biotin was taken up by control vector-treated cardiomyocytes, but only 2% was found in *Yulink* KD cardiomyocytes (Fig. 5d). In the immunofluorescence assay, in which 15d-PGJ2-biotin was stained with streptavidin-Alexa Fluor647 (red), and nuclei were stained with Hoechst 33342 (blue), red signals were observed in the cytoplasm and nuclei of control cardiomyocytes, while no red signals were detected in *Yulink* KD cardiomyocytes (Fig. 5d). These findings indicated that *Yulink* KD block the ligands of PPARγ into cells and result in a decreased nuclear import of PPARγ.

### PPARγ regulates *Serca2* expression in mouse HL-1 cardiomyocytes

**Fig. 6:**
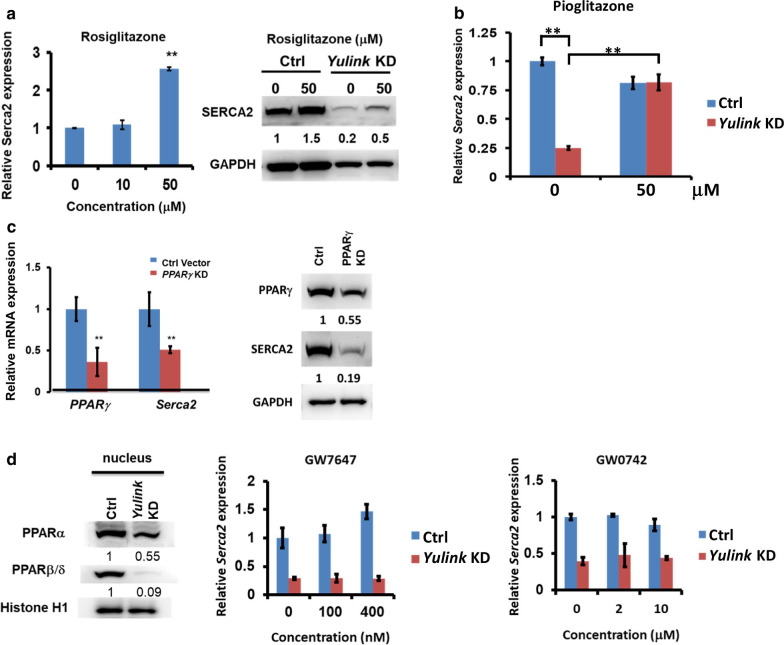
Yulink regulated *Serca2* expression through the PPARγ pathway. **a** Treatment with 50 μM rosiglitazone for 6 h rescued and enhanced *Serca2* expression at mRNA and protein levels in *Yulink* KD cardiomyocytes by qPCR (left panel) and Western blot (right panel). *Serca2* mRNA levels normalized to values in cells treated with Ctrl vector. (n = 3, **p < 0.01, Student’s *t* test). Protein levels normalized to internal control, GAPDH. **b** Treatment with 50 mM pioglitazone (PPARγ agonist) for 12 h increased *Serca2* mRNA expression in *Yulink* KD cardiomyocytes, as shown by qPCR. Data for cardiomyocytes treated with Ctrl vector and *Yulink* KD cardiomyocytes are shown in blue and red, respectively. Before pioglitazone treatment, *Serca2* expression was decreased in *Yulink* KD cardiomyocytes (n = 3, **p < 0.01, Student’s *t* test). Pioglitazone increased relative *Serca2* expression in *Yulink* KD cardiomyocytes from 0.25 to 0.82 (n = 3, **p < 0.01, Student’s *t* test). In Ctrl vector-treated cardiomyocytes, the expressions of *Serca2* before and after pioglitazone were 1 and 0.81, respectively (n = 3). **c** Gene expression and protein levels in *PPARγ*-shRNA KD cardiomyocytes and cells treated with Ctrl Vector were quantified by qRT-PCR and Western blot, respectively. The mRNA expressions of *PPARγ* and *Serca2* were significantly lower in *PPARγ*-shRNA KD cardiomyocytes (0.36 and 0.51, respectively) compared to values of 1 in cells treated with Ctrl vector (n = 3, ** *p* < 0.01, Student’s *t* test). Decreased total cellular PPARγ and SERCA2 protein levels were also observed in *PPARγ*-shRNA KD cardiomyocytes; values normalized to GAPDH. **d** KD of *Yulink* resulted in obvious decreases in nuclear levels of PPARα and PPARβ/δ (left panel, n = 3, ** *p* < 0.01, Student’s *t *test), but treatment with the PPARα agonist GW7647 (middle panel) or PPAR β/δ agonist GW0742 (right panel) did not statistical increase *Serca2* expression in Control vector-treated or *Yulink* KD cardiomyocytes (n = 3)

To examine whether the Yulink regulate Serca2 expression through PPARγ pathway, *Yulink* KD cardiomyocytes were treated with 50 μM rosiglitazone (a PPARγ agonist) for 6 h. As showed in Fig. 6a, the relative *Serca2* mRNA expressions were 1.09 and 2.57 in the presence of 10 and 50 μM rosiglitazone, respectively, compared to the control in the absence of the rosiglitazone. In addition, the SERCA2 protein level could be enhanced to 1.5 folds in control vector-treated cardiomyocytes by 50 μM rosiglitazone treatment for 6 h. Furthermore, in *Yulink* KD cardiomyocytes, the decreased expression of *Serca2* can be rescued from 0.2 to 0.5 folds (Fig. 6a). In parallel, treatment with another PPARγ agonist pioglitazone (50 μM) for 12 h almost completely rescued *Serca2* mRNA expression (Fig. 6b). PPARγ agonists enhanced the SERCA2 expression in the control- and *Yulink* KD cardiomyocytes, suggest that the down regulation of Yulink resulted in a decreased SERCA2 expression may through PPARγ pathway.

To confirm the transcription factor *PPARγ* involved in the *Serca2* expression in cardiomyocytes. HL-1 cells were transduced with lentivirus expressing shRNA against *PPARγ*. Quantitative PCR and western blotting analysis of the transduced cells confirmed a *PPARγ* knockdown efficiency of ~ 70% (Fig. 6c). The relative mRNA and protein levels of *Serca2* were also significantly reduced in these *PPARγ* KD cells (Fig. 6c). These results confirmed that the regulation of the *Serca2* expression by *Yulink* is mediated via PPARγ in HL-1 cardiomyocytes.

We also observed decreased levels of PPARα and PPARβ/δ in *Yulink* KD cardiomyocytes (Fig. 6d). Treatment with PPARα and PPARβ/δ agonists, GW7647 and GW0742, however, did not increase *Serca2* expression in control or *Yulink* KD cardiomyocytes (Fig. 6d), suggesting that PPARα or PPARβ/δ does not involved in *Serca2* expression. Therefore, these results highlight the involvement of *Yulink* with PPARγ in regulating *Serca2* expression in HL-1 cardiomyocytes.

PPARγ has been shown to bind directly to the PPAR response element in the promoter of the SERCA2 gene of pancreatic islet cells [16]. In this study, KD of *PPARγ* resulted in significant decreases in the expression of *Serca2* mRNA and protein in both normal and *Yulink* KD cardiomyocytes. Down-regulation of *Yulink* resulted in a significant reduction in PPARγ DNA binding activity and protein level in the nuclei, demonstrating the role of PPARγ in *Yulink*-mediated transcriptional regulation of *Serca2*. Additionally, PPARγ agonists were found to enhance the expression of *Serca2* in both normal and *Yulink* KD cardiomyocytes. But, treatment with PPARα or PPARβ/δ agonists did not protect against the *Yulink* KD-induced reduction of SERCA2, suggesting the specificity and dependence on PPARγ.

### *PPARγ* over-expression rescued the phenotypes of *Yulink* KD cells

In order to study whether the *PPARγ* over-expression (OE) rescued the phenotypes of *Yulink* KD cells, control (Ctrl) or PPARγ plasmid with BFP as expression marker were transfected into *Yulink* KD cells, and we then analyzed Ca^2+^ cycling. As compared to *Yulink* KD control cells, the intracellular Ca^2+^ cycling waveforms became normal, when these KD cells were over-expressed with *PPARγ* (Fig. 4a). In addition, the results of Ca^2+^ sparks analysis were consistent with the increase of Ca^2+^ transient amplitudes (3.2 ± 0.3 for *PPARγ*-OE, blue bar, vs. 1.9 ± 0.35 for Ctrl, black bar) (Fig. 4a). The cells with *PPARγ*-OE also exhibited a decrease of the percentages of irregular Ca^2+^ transients (10 ± 2% for *PPARγ*-OE, blue bar, vs. 27 ± 3% for Ctrl, black bar) and the reduction of the Ca^2+^ decay rate (450 ± 51 ms for *PPARγ*-OE, blue bar, vs. 605 ± 47 ms for Ctrl, black bar) (Fig. 4a). The level of Ca^2+^ transient amplitudes, the percentages of irregular Ca^2+^ transients, and the Ca^2+^ decay (Tau) rate were all similar between Ctrl (Fig. 3b) and over-expression of *PPARγ* cells. These data indicate that the *PPARγ*-OE rescued the phenotypes of *Yulink* KD cells, suggesting involvement of *Yulink* with PPARγ in regulating *Serca2* expression in HL-1 cardiomyocytes.

### *SERCA2* over-expression rescued the phenotypes of *Yulink* KD cells

In order to examine whether the *SERCA2* over-expression (*SERCA2*-OE) rescued the phenotypes of *Yulink* KD cells, control vector (Ctrl) or *SERCA2* plasmids were electroporated into *Yulink* KD cells with Neon electroporation system. As compared to *Yulink* KD control cells, the intracellular Ca^2+^ cycling waveforms became normal, when these KD cells were over-expressed with *SERCA2* (Fig. 4b). In addition, the results of Ca^2+^ sparks analysis were consistent with the increase of Ca^2+^ transient amplitudes (3.2 ± 0.45 for *SERCA2*-OE, gray bar, vs. 2.02 ± 0.3 for Ctrl, black bar) (Fig. 4b). The cells with *SERCA2*-OE also exhibited a decrease of the percentages of the irregular Ca^2+^ transients (11 ± 3% for *SERCA2*-OE, gray bar, vs. 32 ± 5% for Ctrl, black bar) and the reduction of the Ca^2+^ decay rate (412 ± 50 ms for *SERCA2*-OE, gray bar, vs. 635 ± 78 ms for Ctrl, black bar) (Fig. 4b). The level of Ca^2+^ transient amplitudes, the percentages of irregular Ca^2+^ transients, and the Ca^2+^ decay (Tau) rate were all similar between Ctrl (Fig. 3b) and over-expression of *SERCA2* cells. These data indicate that the *SERCA2*-OE rescued the phenotypes of *Yulink* KD cells, suggesting a specific control for SERCA2 expression by *Yulink* in regulating calcium cycling in HL-1 cardiomyocytes.

### *Yulink* KD induces irregular Ca^2+^ cycling in human cardiomyocytes derived from iPSC

**Fig. 7:**
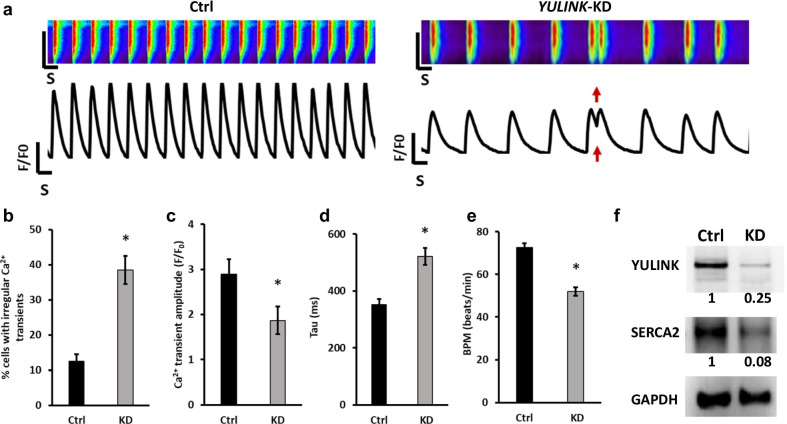
Assessment of arrhythmia and irregular Ca^2+^ regulation in *YULINK* KD iPSC-derived human cardiomyocytes. **a** Representative Ca^2+^ scans and spontaneous Ca^2+^ transient in control and *YULINK* KD iPSC-derived human cardiomyocytes. Red arrows indicate abnormal arrhythmia-like Ca^2+^ waveforms observed in *YULINK* KD cardiomyocytes. **b** Percentages of the irregular Ca^2+^ transients in control and *YULINK* KD iPSC-derived human cardiomyocytes (n = 40). **c** Bar graph comparison of Ca^2+^ transient amplitude of human iPSC-derived cardiomyocytes for control and *YULINK* KD confirmed that *YULINK* KD human cardiomyocytes exhibited a lower Ca^2+^ transient amplitude (n = 40). **d** The time constant for Ca^2+^ decay (Tau) of human iPSC-derived cardiomyocytes for control and *YULINK* KD showed that the time constant for Ca^2+^ decay was significantly larger in *YULINK* KD than in control iPSC-derived human cardiomyocytes (n = 40). **e** Quantification of spontaneous beating rate of control and *YULINK* KD human cardiomyocytes (n = 40). (*p < 0.05, Student’s *t* test). **f** Human cardiomyocytes were subjected to *YULINK* KD using *YULINK*-shRNA and the effects on *YULINK* and *SERCA2* expression were assayed by Western blot

To investigate whether the *YULINK* also involves in intracellular Ca^2+^ cycling in human cardiomyocytes, we analyzed Ca^2+^ cycling in human iPSC-derived cardiomyocytes using fluorescent Ca^2+^ dye Rhod-2 AM. Compared to control, the *YULINK* KD iPSC-derived human cardiomyocytes showed defective intracellular Ca^2+^ cycling with significant higher arrhythmia-like waveforms (38.4% vs. 12.5% for control) (red arrows in Fig. 7a, b), indicative of the Ca^2+^ transient irregularities. In addition, smaller Ca^2+^ transient amplitudes (1.87 ± 0.31 for *YULINK* KD vs. 2.89 ± 0.34 for control) (Fig. 7c) and slower Ca^2+^ decay rate (521 ± 30 ms for *YULINK* KD vs. 351 ± 21 ms for control) (Fig. 7d) were also found. These observations of the Ca^2+^ transients of human iPSC-derived cardiomyocytes are consistent with the results using mouse HL-1 cardiomyocytes, indicating that irregular Ca^2+^ cycling is a feature of cardiomyocytes for mouse and human, when *Yulink* was knockdown.

Furthermore, the contractile function of the control and *YULINK* KD cardiomyocytes was also examined. The cell contraction rate was counted under a phase-contrast microscope, it was found that *YULINK* KD resulted in reduction of spontaneous beating rate, as shown with a reduced beating frequency by 28.3% (Fig. 7e).

To determine the *SERAC2* expression in the *YULINK* KD human cardiomyocytes, western blotting analysis was performed. It was found that there were approximately 90% reduction of *SERCA2* expression in the *YULINK* KD cardiomyocytes (Fig. 7f). These results imply that *YULINK* regulates *SERCA2* expression and intracellular Ca^2+^ cycling in human cardiomyocytes.

## Discussions

In this study, we have demonstrated that *Yulink* is highly conserved in zebrafish, mouse, and human. It was expressed in zebrafish embryo ubiquitously from zygote stage to larval stage and expressed in heart region starting from 1 dpf. The y*ulink* KD morphants in zebrafish exhibited pericardial edema, slower heart rate, and reduced cardiac output. Besides, we also observed that down-regulation of *Yulin*k in the mouse and iPSC-derived human cardiomyocytes exhibited defective intracellular Ca^2+^ cycling. Importantly it was observed that *YULINK*, *PPARγ* or *SERCA2* over-expression specifically rescued the phenotypes of *Yulink* KD cells. These results thus suggest that *Yulink* is involved in intracellular Ca^2+^ cycling in cardiomyocytes.

Ca^2+^ cycling is crucial for excitation–contraction coupling of cardiomyocytes and is essential in the electrical signaling of cardiomyocytes. Abnormal Ca^2+^ cycling is linked to arrhythmogenesis, which is associated with cardiac disorders and heart failure [10]. KD of *Yulink* in mouse and human cardiomyocytes displayed a decrease in *SERCA2* expression and exhibited higher Ca^2+^ transient irregularities which are accompanied with the triggered arrhythmia, defective intracellular Ca^2+^ cycling with a reduced Ca^2+^ transient amplitudes, and slower Ca^2+^ decay rate. In addition, *YULINK*, *PPARγ* or *SERCA2* over-expression restored these phenotypes of mouse *Yulink* KD cells, indicating that the *Yulink*-shRNA used in our studies was specific and the observed defects in cells were Yulink-dependent.

When SERCA2 was deleted in mouse heart using the tamoxifen-inducible Cre, The Tau of Ca^2+^ transient was increased by ~ 90 to 118% in SERCA2-deficient cardiomyocytes [1, 19]. Additionally, it is known that the decreases in *SERCA2* expression resulted in a diminished Ca^2+^ content in the sarcoplasmic reticulum, which reduced systolic Ca^2+^ release and impairment of myocardial contractility [10]. Therefore, decrease in *Yulink* function may play an important role in susceptibility to heart arrhythmia via impairment of cardiac SERCA2 activity.

Our studies indicated that knockdown of *Yulink* resulted in a decrease of nuclear PPARα and β/δ levels in HL-1 cardiomyocytes. Previously, it was reported that PPARs were major executors for modulating homeostasis of glucoses and lipids in heart [3]. Overexpression of PPARα induced several target genes involved in fatty acid utilization and increased fatty acid uptake and oxidation in heart [8]. On the other hand, PPARα null mice exhibited an increase in glucose transporter expression and glucose uptake [5, 23]. Mice with cardiac-specific deletion of PPARβ/δ were shown to exhibit severe impairments in myocardial fatty acid oxidation gene expression and increased cardiac lipid accumulation [6]. Therefore, the decreased nuclear PPARα and PPARβ/δ levels observed in *Yulink* KD cardiomyocytes would suggest that Yulink may has other important functions in the maintenance of glucose and lipid homeostasis via regulating PPARα or PPARβ/δ activities.

Some natural ligands can bind PPARγ, like unsaturated fatty acid, 15-Hydroxyeicosatetraenoic acid, 9- and 13-hydroxyoctadecadienoic acid or PGJ2 [18]. It is unknown which ligand is required for cardiomyocytes and entry into cells via Yulink. Transcriptional activity of PPARγ is regulated primarily by ligand binding [33]and 15d-PGJ2 is thought to be the most potent endogenous ligand for PPARγ [9, 36]. The 15d-PGJ2 was detected in exosomes; and exosomes were internalized and accumulated in an endosomal compartment [30]. Here, we used 15d-PGJ2-biotin as PPARγ ligand and KD of *Yulink* blocked the entry of ligand into cells. Therefore, *Yulink* KD resulted in a reduced nuclear import of PPARγ, a decreased SERCA2 expression, and abnormal Ca^2+^ cycling in the mouse HL-1 cardiomyocytes. Furthermore, it was reported that PPARγ bound a PPAR response element in the − 259 bp proximal region of SERCA2 promoter in pancreatic cells, based on luciferase reporter assay, EMSA, and chromatin immunoprecipitation [16]. In addition, the *SERCA2* promoter region of rabbit, rat, mouse and human all displayed the PPARγ binding site [16]. However, there was no direct evidence to demonstrate a binding of PPARγ in cardiomyocytes. We would await for studies in the future to investigate in details how Yulink modulates uptake of ligand for PPARγ, thus regulating *SERCA2* in cells. In addition to PPARγ signaling, other pathways might be impaired when *Yulink* was knockdown, indicating the need for future studies to validate detailed mechanism.

## Conclusions

Deficiency of *yulink* caused cardiac dysfunction in zebrafish, manifested by pericardial edema, decreased beating rate and cardiac output. In addition, down regulation of *Yulink* in mouse and human iPSC-derived cardiomyocytes resulted in greater Ca^2+^ transient irregularities including defective intracellular Ca^2+^ cycling, reduced Ca^2+^ transient amplitudes, and slower Ca^2+^ decay rate, thereby triggering arrhythmia. Besides, *YULINK, PPARγ* or *SERCA2* over-expression rescued these phenotypes of mouse *Yulink* KD cells. Mechanistically, deficiency of *Yulink* reduced expression of cardiac SERCA2 mediated by PPARγ nucleus entry.

Importantly, our results highlight the involvement of *Yulink* with PPARγ in regulating *SERCA2* expression, which may shed light on many debates about the risks and benefits of PPARγ agonists in clinical use. Finally, this *Yulink* gene was first identified through comparative evolutionary genomics analysis and reverse screening involving genetic knockdown in zebrafish. The strategies of using the initial observations in zebrafish for the identification of biological functions in mouse HL-1 cardiomyocytes and human iPSC-derived cardiomyocytes provide new paradigm for the study of diseases mechanisms of other specific/novel genes.

## Supplementary Information

**Additional file 1:** Video.

**Additional file 2: Fig. S1.** Prediction of secondary structure for YULINK. **Fig. S2.** Similar phenotypes of *yulink* knockdown were observed in embryos after microinjection with MO that targeted against the splicing site or start site. **Fig. S3.** The expressions of heart rate-related genes were reduced in *yulink* KD morphants. **Fig. S4.** The phenotypes of the morphants were rescued via over-expression of *Yulink*.

## Data Availability

All data generated or analyzed during this study are included in this article.
