# Supplementary material for: Yulink, predicted from evolutionary analysis, is involved in cardiac function
Source: J Biomed Sci. 2021 Jan 11;28:7. doi: 10.1186/s12929-020-00701-7 (PMC7798328; doi:10.1186/s12929-020-00701-7)

**Fig. S1. Prediction of secondary structure for YULINK.** The pattern of secondary structure within YULINK from amino acid 1 to 480 was predicted using Jpred4 (http://www.compbio.dundee.ac.uk/jpred). The green arrows represent β strands. The Jpred4 score for each amino acid was shown: high values mean high confidence of the prediction for secondary structure for that position. The labels for WD40 repeats #1–#4 and A–C are the same as that shown in Fig. 1C.


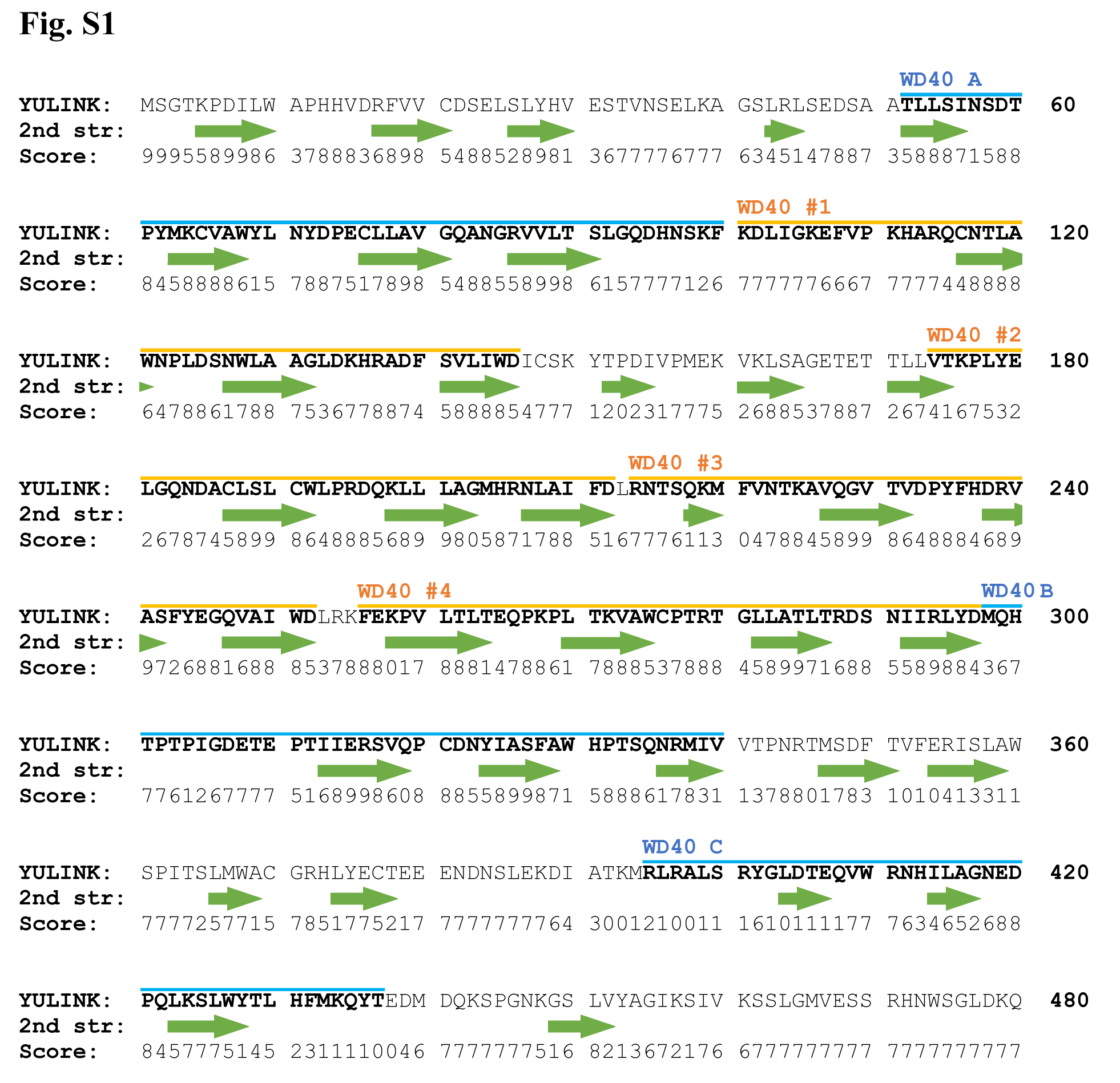


**Fig. S2. Similar phenotypes of *yulink* knockdown were observed in embryos after microinjection with MO that targeted against the splicing site or start site.** A, WT embryo. B, morphant with MO against the splicing site of yulink gene. C, Morphant with MO against translational start site of yulink gene. After microinjection of these two MOs, morphants displayed small eyes, a small head, abnormal vessel formation, and pericardial edema (indicated as arrows) at 3dpf, similar to those found in Fig. 2B.


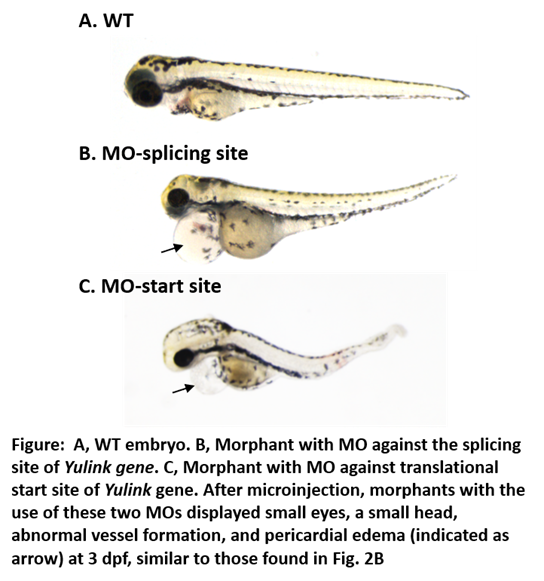


**Fig. S3. The expressions of heart rate-related genes were reduced in *yulink* KD morphants.** A, Sequences of qPCR primers used. B, Expression of *serca2a* was decreased in *yulink* KD morphants at 3 dpf (30 larvae for each group, n = 3, ***p* < 0.01, Student’s *t*-test). C, Expressions of *hcn4,* *a1G, a1Ha, a1Hb, a1la and a1lb* were decreased significantly in *yulink* KD morphants (30 larvae for each group, n = 3, ***p* < 0.01, Student’s *t*-test).

**
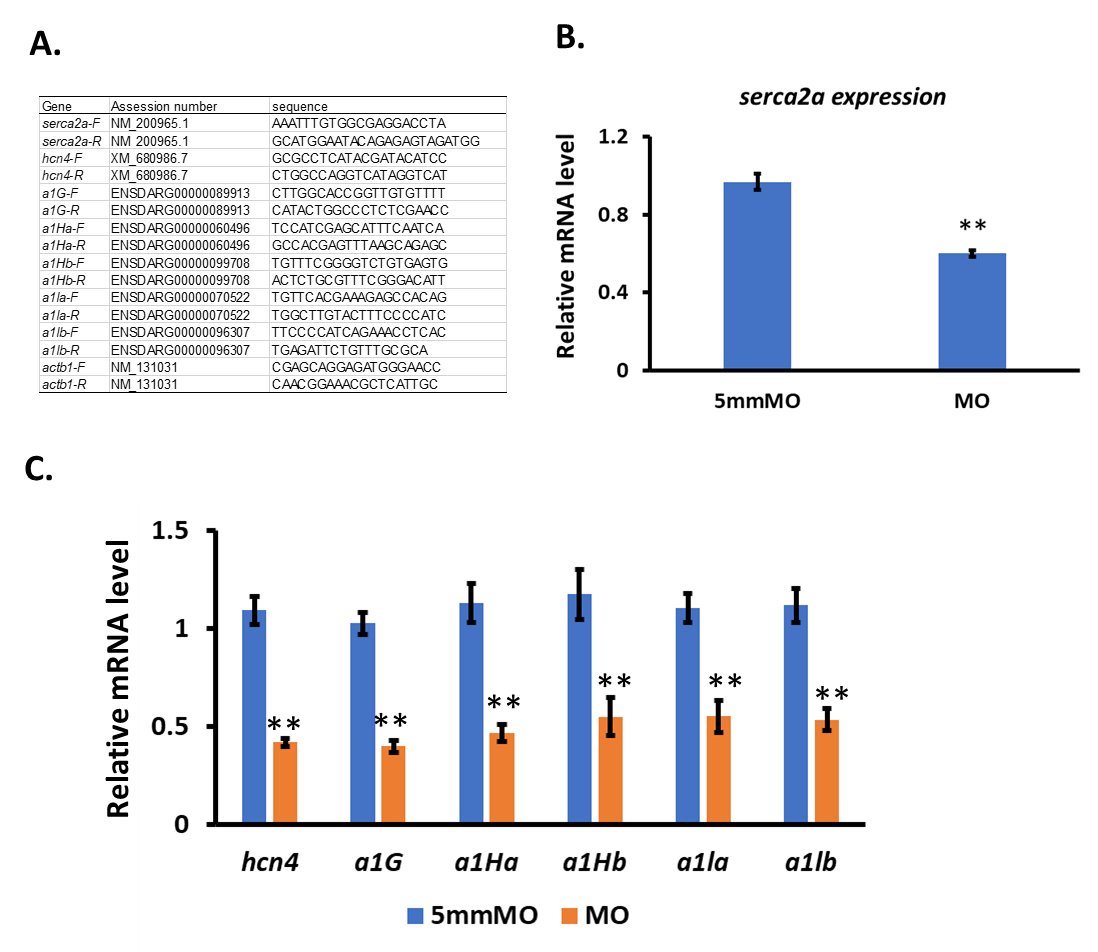
**

**Fig. S4. The phenotypes of the morphants were rescued via over-expression of *Yulink***. For rescue experiments, the *yulink*-MO (4.6 ng/embryo) was either injected alone into embryos or co-injected with the mouse *Yulink* mRNA (*mYulink* mRNA, 200 pg/embryo) or the control *β-gal* mRNA (200 pg/embryo). After injection of *yulink*-MO alone (n = 62), 65.8 % of morphants had a severe phenotype and 18.6 % a moderate phenotype. Co-injection of *yulink*-MO with a control *β-gal* mRNA (n = 45) led to severe and moderate morphant phenotype percentages of 62.2 % and 15.6 %, respectively, similar to levels produced by *yulink*-MO alone. In contrast, when *yulink*-MO was co-injected with the mouse *Yulink* mRNA (n = 81), a rescue effect was observed, and the percentages of severe and moderate phenotypes became 38.7 % and 30.6 %, respectively.


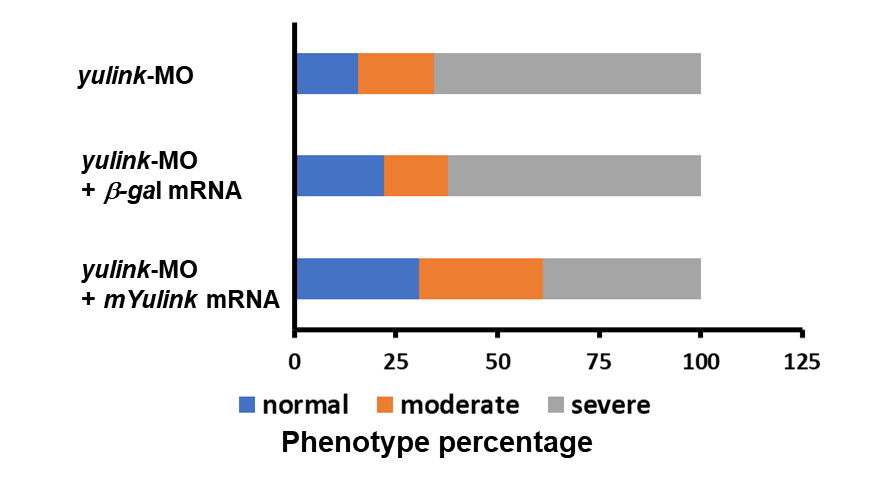

Supplement: Supplementary file 2 — Additional file 2: Fig. S1. Prediction of secondary structure for YULINK. Fig. S2. Similar phenotypes of yulink knockdown were observed in embryos after microinjection with MO that targeted against the splicing site or start site. Fig. S3. The expressions of heart rate-related genes were reduced in yulink KD morphants. Fig. S4. The phenotypes of the morphants were rescued via over-expression of Yulink. [file 12929_2020_701_MOESM2_ESM.docx]
